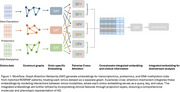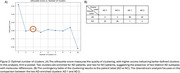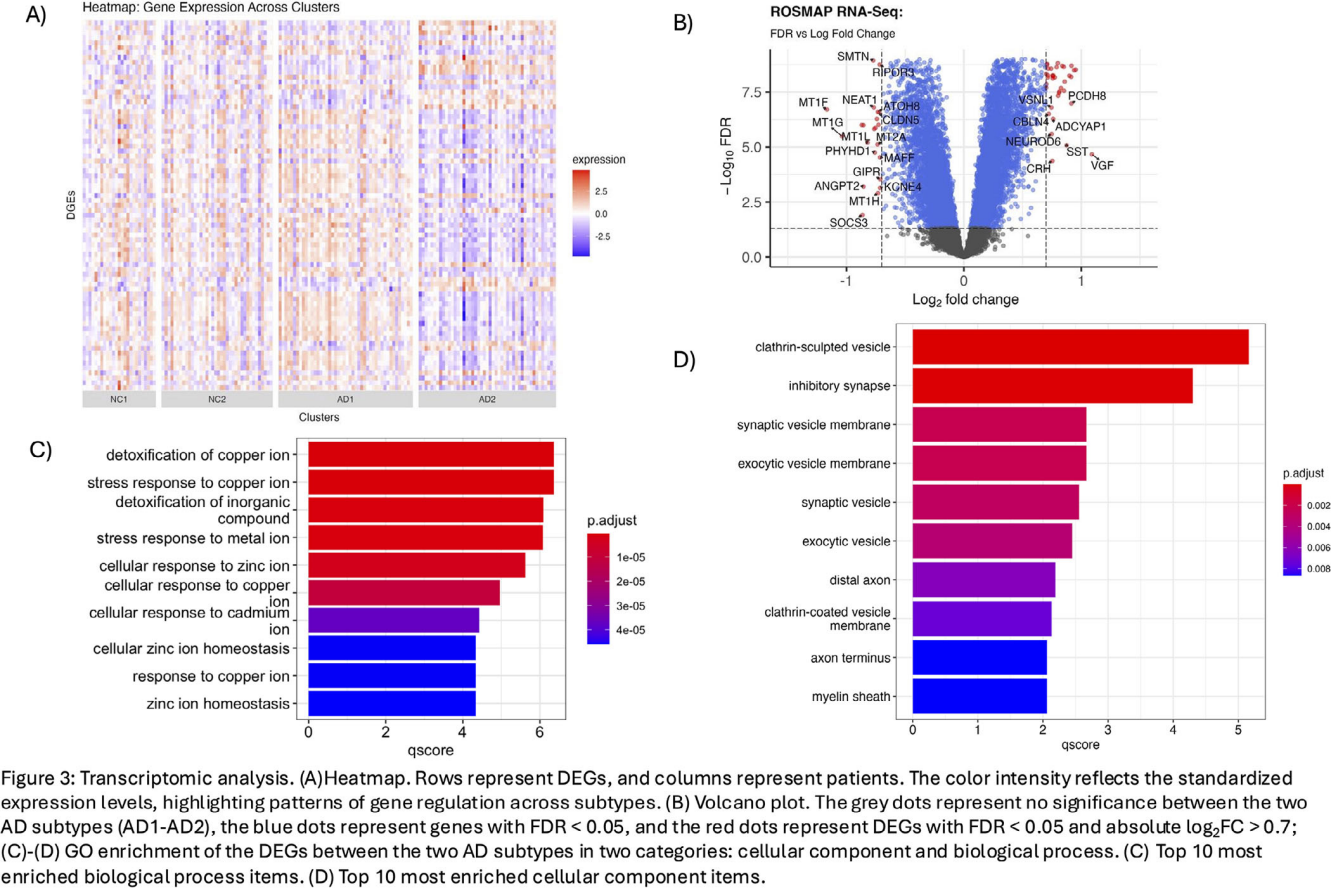# Integrative Multi‐Omics Approach with Graph Attention Network and Cross‐Attention to Uncover Alzheimer's Disease Subtypes

**DOI:** 10.1002/alz70856_107088

**Published:** 2026-01-08

**Authors:** Ziyan Song, Xiaoqing Huang, Jiahui Liu, Junxiang Chen, Travis S Johnson, Jie Zhang, Kun Huang

**Affiliations:** ^1^ Indiana University, School of Medicine, Indianapolis, IN, USA; ^2^ Indiana University, Fairbanks School of Public Health, Indianapolis, IN, USA

## Abstract

**Background:**

Distinguishing Alzheimer's Disease (AD) subtypes can improve disease diagnosis, treatment, and management. This study uses Graph Attention Networks (GAT) and a cross‐attention algorithm to integrate multi‐omics data and characterize distinct AD subtypes.

**Method:**

Three omics datasets, including transcriptomics, proteomics, DNA methylation, and clinical information from 156 Religious Orders Study and Rush Memory and Aging Project (ROSMAP) patients and controls, were integrated using a Graph Attention Network (GAT) and a cross‐attention mechanism. GAT encoders generated embeddings for each omics graph, which were integrated via pairwise cross‐attention and combined with clinical data through projection layers. A multi‐task loss combining cross‐entropy and reconstruction losses was used for training, yielding integrated embeddings representing the molecular complexity of AD (Figure 1). Pseudotime for all patients was calculated using the Partition‐based Graph Abstraction (PAGA) method to compare disease progression trajectories across subtypes identified by KMeans. Differentially expressed genes (DEGs) and clinical differences between AD‐enriched clusters were evaluated to characterize AD subtypes, and gene set enrichment analysis identified molecular functions, biological processes, and cellular components enriched among DEGs.

**Result:**

Four clusters were identified: two enriched in controls and two in AD (Figure 2). This study focuses on comparing the two AD subtypes. Pseudotime analysis showed significant trajectory differences among all clusters, particularly between the AD subtypes (adjusted *p* < 0.01), indicating distinct disease trajectories. Demographic factors age, sex, and APOE status, showed no significant differences between the two subtypes. Cognitive status differed significantly, with one AD subtype including more patients with mild cognitive impairment (MCI). Additionally, 75 DEGs between the two subtypes were identified (FDR < 0.05 and logFC > 0.7), along with 126 significant molecular processes, 3 biological functions, and 38 cell components were identified, with adjusted *p*‐values < 0.1 (Figure 3).

**Conclusion:**

The pipeline integrated multimodal omics data and successfully identified two AD subtypes with distinct disease progression. The 75 DEGs are enriched cellular response to copper ions, hormone activity, and mitochondrial respiratory chain complex I and clathrin‐sculpted vesicles, which we believe play important roles in AD pathogenesis and differentiate the two subtypes.